# Echocardiographically Measured Epicardial Fat Predicts New-onset Atrial Fibrillation after Cardiac Surgery

**DOI:** 10.21470/1678-9741-2019-0388

**Published:** 2020

**Authors:** Ertugrul Emre Gunturk, Mustafa Topuz, Faruk Serhatlioğlu, Hasan Akkaya

**Affiliations:** 1 Ömer Halisdemir University, Cardiology, Niğde, Turkey.; 2 Ömer Halisdemir University, Cardiovascular Surgery, Niğde, Turkey.; 3 University of Health Sciences Adana City Education and Research Hospital, Cardiology Adana, Turkey,

**Keywords:** Coronary Artery Disease, Mammary Arteries, Cardiopulmonary Bypass, Logistic Models, Atrial Fibrilation, Coronary Artery Bypass, Natriuretic Peptide, Brain, Echocardiography

## Abstract

**Objective:**

The current study aims to investigate the role of echocardiographically measured epicardial adipose tissue (EAT) thickness in the prediction of new-onset atrial fibrillation (AF) following coronary artery bypass grafting (CABG) surgery.

**Methods:**

One hundred and twenty-four patients scheduled to undergo isolated on-pump CABG due to coronary artery disease were enrolled to the current study. Patient characteristics, medical history and perioperative variables were prospectively collected. EAT thickness was measured using transthoracic echocardiography (TTE). Any documented episode of new-onset postoperative AF (POAF) until discharge was defined as the study endpoint. Fortyfour participants with POAF served as AF group and 80 patients without AF served as Non-AF group.

**Results:**

Two groups were similar in terms of baseline echocardiographic and laboratory findings. In laboratory findings, the groups were similar in terms of the studied parameters, except N-terminal pro-brain natriuretic peptide (NT Pro-BNP), which was higher in AF group than in Non-AF group (*P*=0.035). The number of left internal mammary artery (LIMA) grafts was not different in both groups. AF group had higher cross-clamp (CC) and cardiopulmonary bypass (CPB) times than Non-AF group (*P*=0.01 and *P*<0.001). In multivariate logistic regression analysis, EAT was found an independent predictor for the development of POAF (OR 4.47, 95% CI 3.07-5.87, *P*=0.001).

**Conclusion:**

We have shown that EAT thickness is associated with increased risk of AF development and can be used as a prognostic marker for this purpose.

**Table:** 

Abbreviations, acronyms & symbols				
**AF**	**= Atrial fibrillation**	** **	**LAVI**	**= Left atrial volume index**
**CABG**	**= Coronary artery bypass grafting**		**LIMA**	**= Left internal mammary artery**
**CC**	**= Cross-clamp**		**NT Pro-BNP**	**= N-terminal pro-brain natriuretic peptide**
**CI**	**= Confidence interval**		**OR**	**= Odds ratio**
**CT**	**= Computed tomography**		**POAF**	**= Postoperative atrial fibrillation**
**CPB**	**= Cardiopulmonary bypass**		**ROC**	**= Receiver operating characteristic**
**EAT**	**= Epicardial adipose tissue**		**ROS**	**= Reactive oxygen species**
**ECG**	**= Electrocardiography**		**SPSS**	**= Social Package for the Social Sciences**
**LA**	**= Left atrium**		**TTE**	**= Transthoracic echocardiography**

## INTRODUCTION

Postoperative atrial fibrillation (POAF) occurs in up to 50% of patients undergoing cardiac surgery and represents the most common postoperative arrhythmic complication^[1]^. This is clinically important, since POAF increases the risk of short- and long-term morbidity and mortality and prolongs hospital stay^[2]^. Clinical variables such as age, hypertension, male gender, left ventricular dysfunction, history of atrial fibrillation (AF) and intraoperative surgical factors are well-documented risk factors and affect the frequency of POAF^[3]^.

Epicardial adipose tissue (EAT) is located between myocardium and visceral pericardium and has direct contact with the myocardium^[4]^. Previous clinical studies have shown a strong relationship between EAT and AF development^[5,6]^. It is well known that one of the major physiopathology of AF development is atrial structural changes due to atrial fibrosis^[7]^. Thus, as a rich source of adipocytokines, EAT has pro-fibrotic effects on the atria because its secretions directly affect the atrial myocardium^[8]^.

Preoperative identification of which patients at high risk for POAF after coronary artery bypass grafting (CABG) is clinically important, since POAF increases the risk of short- and longterm morbidity and mortality and prolongs hospital stay after any surgery, as indicated above. In addition, the most effective preventive methods, such as amiodarone infusion or atrial pacing, are not cost-effective. Therefore, we aimed to investigate the relationship between EAT thickness measured by transthoracic echocardiography (TTE) and POAF development in patients undergoing CABG surgery.

## METHODS

One hundred and twenty-four patients who underwent elective CABG surgery at our cardiac surgery clinic between January 2017 and January 2018 were included in the current study. Demographic characteristics, medical history, intraoperative variables and postoperative status of the study patients were recorded. Patients with a history of any cardiac surgery, simultaneous valve surgery, a history of arrhythmias or antiarrhythmic drug use, systemic and any inflammatory disease, thyroid disorders, chronic renal and liver disease were excluded from the study. This study was approved by local ethics committee, also complied with the Declaration of Helsinki, and signed; written informed consent was obtained from all patients.

### Biochemical Analysis

Blood samples were obtained perioperatively and appropriate tubes were collected for further analysis. The measurements were performed by an automatic blood counter (Roche Diagnostics, Indianapolis, IN, USA) with commercially available kits. The N-terminal pro-brain natriuretic peptide (NT Pro-BNP) levels were assessed by using immunoturbidimetry (Beckmann Assay 360, Bera, California, USA). Hematological parameters were measured from tripotassium ethylenediaminetetraacetic acidbased anticoagulated blood samples and assessed by a Sysmex K-1000 (Block Scientific, Bohemia, NY, USA) autoanalyzer within 30 minutes of sampling.

### Transthoracic Echocardiography

**Fig. 1:**
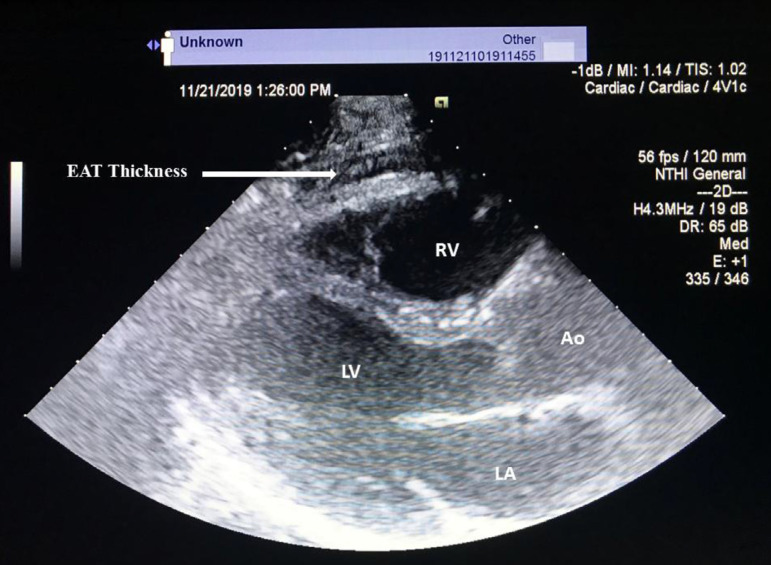
EAT was identified as an echo-free space between external myocardium and visceral pericardium from the parasternal long-axis view and was measured perpendicularly in front of the right ventricular free wall at end-diastole; AO=aorta; LA=left atrium; LV=left ventricle; RV=right ventricle

Standard transthoracic echocardiographic evaluation was performed in all patients before surgery (Vivid S6, GE Healthcare, USA). To standardize the measurement axis, we used the aortic annulus as an anatomical reference. EAT was defined as an echo-free region between the right ventricular outer wall and the visceral pericardium in parasternal long axis imaging^[9]^. The static image of EAT thickness was zoomed for better visualization and accurate measurement (Figure 1). Measurements were made throughout 3 consecutive cardiac cycles and the average value was taken. Interobserver correlation coefficients were 0.92, whereas the intraobserver correlation coefficients were 0.95 (the mean intraobserver absolute difference in EAT measurements was 0.2±0.3 mm and the coefficient of variation was 1.4%).

### Intra/Postoperative Properties

All patients were operated with standard median sternotomy and cardiopulmonary bypass pump. According to the patient’s clinical and vascular status, left anterior mammary artery and/or saphenous grafts were used for anastomoses. All patients were transferred to the intensive care unit after surgery. Patient vital signs were evaluated every 2 hours. To eliminate the secondary causes of new-onset AF, we evaluated all postoperative fluid-electrolyte balances of patients and any electrolyte deficiency was treated by replacement. We also started the beta-blocker treatment on the 1^st^ postoperative day in all patients.

### Electrocardiographic Monitoring

The patients’ cardiac rhythm was continuously followed-up in the cardiac surgery intensive care unit. In the normal patient room, patients’ cardiac rhythm was recorded by central telemetry. Electrocardiography (ECG) of all patients was performed daily. A new ECG was performed when patients described a symptom such as palpitations, shortness of breath, dizziness, and chest pain. During the follow-up period, arrhythmia episodes that deteriorated hemodynamics or lasted more than 20 minutes at one time or lasted more than 60 minutes at 24-hour followup were defined as POAF. Patients who developed AF were treated with intravenous amiodarone. Electrical cardioversion was performed in patients hemodynamically impaired and unresponsive to amiodarone treatment.

### Statistical Analysis

SPSS statistical software (version 15.0, SPSS, USA) was used for statistical analysis. Continuous variables were expressed as mean ± SD or interquartile range in the presence of abnormal distribution, and categorical variables as percentages. Comparisons between groups of patients were made by the chisquare test for categorical variables, independent samples t-test for normally distributed continuous variables, and Mann-Whitney U test when the distribution was skewed. The correlation of EAT thickness with other study parameters was evaluated either via Pearson or Spearman correlation tests. Using regression analysis, other possible confounding covariables were adjusted. Receiver operating characteristic (ROC) analysis was used to determine a cutoff value for EAT thickness. A *P*-value <0.05 was accepted as statistically significant.

## RESULTS

Baseline characteristics, echocardiographic and laboratory findings, and surgery characteristics of the two groups are listed in Table 1. There were 44 patients (mean age 65.5±5.4 years, 28 men) in the AF group and 80 patients (mean age 64.8±7.1 years, 50 men) in the Non-AF group. Pre-treatments before the index hospitalization, risk factors for coronary artery disease such as smoking, diabetes mellitus, hypertension and anamnesis were similar between the two groups (for all *P*>0.05). Groups were also similar regarding the prevalence of chronic obstructive pulmonary disease and functional status before surgery, which can affect the postoperative situation.

**Table 1:** Demographic, echocardiographic and laboratory characteristics of the study groups.

		AF group (n=45)	Non-AF group (n=80)	*P*-value*
Age, years, mean±SD		65.5±5.4	64.8±7.1	0.31
Male gender, n		28 (52.2%)	50 (62.5%)	0.97
Smoking, n		25 (55.5%)	41(51.2%)	0.64
BMI, kg/m^2^, mean±SD		27.3±1.9	27.1±2.3	0.51
DM, n		18 (40.0%)	31(38.7%)	0.89
HT, n		33 (73.3%)	53 (66.2%)	0.41
COPD, n		5 (11.1%)	9 (11.2%)	0.98
Stroke, n		3 (6.7%)	6 (7.5%)	0.86
Previous MI, n		14 (31.1%)	30 (37.5%)	0.47
NYHA class (stages 1/2/3)		0/35/10	3/59/18	0.41
Heart rate, bpm		75.1±9.5	73.4±9.8	0.36
Laboratory characteristics	Glucose, mg/dl	97.6±9.7	98.4±9.5	0.51
	Creatinine, mg/dl	1.1±0.14	1.09±0.17	0. 6
	Sodium	140.1±2.8	140.7±2.7	0.97
	Potassium	4.4±0.6	4.5±0.5	0.15
	C-reactive protein, mg/dl	3.66±1.09	3.75±0.89	0.47
	Total-C, mg/dl	208.8±24.6	204.7 ±20.0	0.31
	LDL-C, mg/dl	135.7±21.5	132.2±19.4	0.32
	HDL-C, mg/dl	37.5±3.5	39.0±5.1	0.089
	TG, mg/dl	175.7±33.6	168.0±38.9	0.26
	TSH	2.6±0.57	2.6±0.49	0.94
	NT Pro-BNP, mg/dl	110.2±23.5	101.0±23.1	0.035
Surgery characteristics	Elective, n	37 (82.2%)	64 (80.0%)	0.76
	LIMA graft, n	36 (80.0%)	67 (83.7%)	0.59
	CC time, min	55.1±10.5	48.7±9.4	0.001
	CPB time, min	85.1±8.1	75.1±9.2	<0.001
	Total graft number, n	2.95±0.45	3.01±0.53	0.53
Echocardiography	LVEF, %	55.1±5.9	55.8±5.9	0.45
	LA diameter, mm	39.2±2.5	36.0±3.4	<0.001
	LA volume index	28.3±3.5	24.9±3.0	<0.001
	EAT thickness, mm	7.28±0.57	6.42±0.35	<0.001
Drugs	Beta-blockers, n	23 (51.1%)	39 (48.7%)	0.80
	ACE/ARB inhibitors, n	12 (26.7%)	29 (36.2%)	0.27
	Diuretics, n	25 (55.5%)	56 (70.0%)	0.10
	CCB, n	32 (71.1%)	61 (76.2%)	0.50
	Statins, n	22 (48.9%)	36 (45.0%)	0.67

ACE=angiotensin-converting enzyme; ARB=angiotensin II receptor blockers; BMI=body mass index; CCB=calcium channel blockers; COPD=chronic obstructive pulmonary disease; CRP=C-reactive protein; DM=diabetes mellitus; EAT=epicardial adipose tissue; HDL-C=high-density lipoprotein cholesterol; HT=hypertension; LA=left atrium; LDL-C=low-density lipoprotein cholesterol; LIMA=left internal mammary artery; LVEF=left ventricular ejection fraction; NYHA=New York Heart Association; SD=standard deviation; TG=triglyceride; Total-C=total cholesterol; TSH=thyroid stimulating hormone.

* A *P*<0.05 was accepted as significant.

In laboratory findings, the groups were similar according to the study parameters, except NT Pro-BNP, which was higher in AF group than in Non-AF group patients (110.2±23.5 mg/dl *vs*. 101.0±23.1 mg/dl, *P*=0.035) (Table 1).

The surgical characteristics of the groups were also listed in Table 1. The number of left internal mammary artery (LIMA) grafts used was not different in both groups. The AF group had a higher cross-clamp (CC) and cardiopulmonary bypass (CPB) times than the Non-AF group (55.1±10.5 min *vs*. 48.7±9.4 min, *P*=0.01 and 85.1±8.1 min *vs*. 75.1±9.2 min, *P*<0.001, respectively) (Table 1).

Both groups have similar left ventricular systolic performances. The mean left ventricular ejection fraction was 55.1±5.9% in AF group and 55.8±5.9% in Non-AF group (*P*=0.45), while left atrial volume index and left atrial diameter were higher in AF group than in Non-AF group (*P*<0.001 for both). In addition, AF group had a higher EAT thickness than Non-AF group (7.28±0.57 mm *vs*. 6.42±0.35 mm, *P*<0.001).

In correlation analysis, mean EAT thickness was significantly correlated with both CC and CPB times (r:0.342, *P*<0.001 and r:0.309, *P*<0.001, respectively). EAT was also well correlated with body mass index (r:0.332, *P*<0.001) and triglyceride level (r:0.294, *P*:0.031). In addition, parameters related with diastolic properties, such as left atrial (LA) diameter and LA volume, were also correlated with EAT thickness (r:0.348, *P*<0.001 and r:0.316, *P*:0.012) (Table 2).

**Table 2:** Correlation analysis of EAT thickness with study parameters in all study patients.

	Correlation coefficient	*P*-value*
Age	0.279	0.054
BMI	0.332	<0.001
Heart rate	0.07	0.83
C-reactive protein	0.155	0.303
NT Pro-BNP	0.11	0.51
TG	0.294	0.031
LDL-C	0.256	0.044
HDL-C	- 0.205	0.057
LA diameter	0.348	<0.001
LA volume index	0.316	0.012
CC time	0.342	<0.001
CPB time	0.309	<0.001

BMI=body mass index; CC=cross-clamp; CPB=cardiopulmonary bypass; EAT=epicardial adipose tissue; HDL-C=high-density lipoprotein cholesterol; LA=left atrium; LDL-C=low density lipoprotein cholesterol; LIMA=left internal mammary artery; SD=standard deviation; TG=triglyceride.

* A P<0.05 was accepted as significant.

**Fig. 2:**
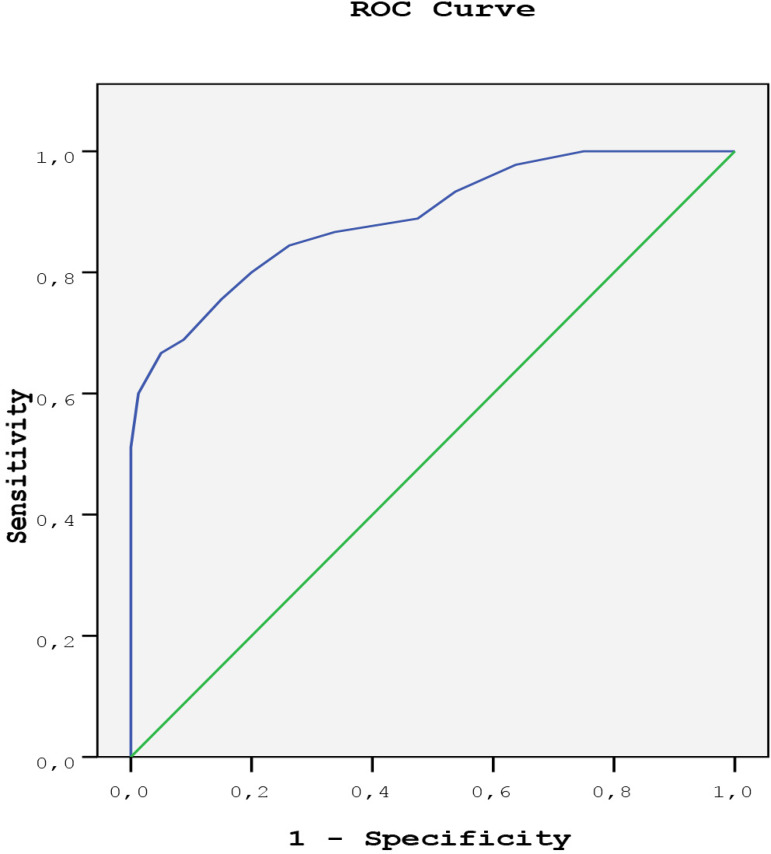
ROC analysis showed 67% sensitivity and 61% specificity for a cutoff value of 0.70 for EAT (P<0.001 area under the ROC curve: 0.890).

The study parameters including NT Pro-BNP, LA diameter and LA volume index (LAVI), EAT, graft number, statin use, CC and CPB times and age were analyzed in a multivariate logistic regression model. The graft number (OR 4.71, 95% CI 2.88-5.76, *P*=0.02), EAT (OR 4.47, 95% CI 3.07-5.87, *P*=0.001), NT Pro-BNP level (OR 1.13, 95% CI 1.02-1.25, *P*=0.014), CC and CPB times (OR 0.73, 95% CI 0.52-0.92, *P*=0.001 and OR 2.07, 95% CI 1.32-3.01, *P*=0.001, respectively) and statin use (OR 1.37, 95% CI 0.88-1.86, *P*=0.03) remained independent predictors for the development of POAF (Table 3). The ROC analysis showed that the best discriminatory level of EAT thickness to predict new-onset POAF was 7.05 mm (67% sensitivity and 61% specificity) (Figure 2). The area under the curve was 0.890 with 95% CI of 0.771-0.978 and a *P*-value of <0.001.

**Table 3:** Univariate and multivariate logistic regression analysis showing parameters associated with POAF.

	Unadjusted OR/95%CI	*P*-value*	Adjusted OR/95%CI	*P*-value*
Age	1.21(0.97-1.51)	0.090	1.22 (1.08-1.48)	0.048
NT ProBNP	1.07(0.94-1.21)	0.295	1.13(1.02-1.25)	0.014
LVEF	0.79(0.51-1.19)	0.266		
LA	2.42(0.80-7.26)	0.115		
LAVI	4.21(3.87-9.25)	0.303		
EAT	3.11(2.07-4.25)	0.003	4.47(3.07-5.87)	0.001
Used graft	5.31(3.77-7.02)	0.03	4.71(2.88-5.76)	0.02
Statin	1.42(0.80-2.26)	0.02	1.37(0.88-1.86)	0.03
LIMA	1.39(0.05-31.57)	0.84		
CC time	0.69(0.52-0.91)	0.001	0.73(0.52-0.92)	0.001
CPB time	2.17(1.33-3.23)	0.013	2.07(1.32-3.01)	0.001

BMI=body mass index; CC=cross-clamp; CI=confidence interval; CPB=cardiopulmonary bypass; EAT=epicardial adipose tissue; LA=left atrium; LAVI=left atrial volume index; LIMA=left internal mammary artery; LVEF=left ventricular ejection fraction; LVMI=left ventricle mass index; OR=odds ratio; POAF=postoperative atrial fibrillation.

* A *P*<0.05 was accepted as significant.

## DISCUSSION

This is the first study in literature that clearly demonstrated the association of EAT measured by preoperative TTE with POAF development in patients undergoing CABG. In the current study, we found that echocardiographically measured EAT thickness by TTE was significantly correlated and predicted new-onset AF in patients undergoing CABG.

POAF is one of the most common complication after CABG surgery and its incidence is still high despite advances in surgical techniques. A number of clinical and demographic factors, such as advanced age, male gender, hypertension, history of AF, chronic obstructive pulmonary disease, heart failure, obesity and renal failure affect POAF development^[10]^. Identifying patients vulnerable to postoperative AF would target those most likely to benefit from aggressive prophylactic intervention.

Mechanisms leading to AF have a complex pathophysiology (remodeling of atrial structure, ion channel functions, electrophysiological mechanisms, etc.) and, as an ectopic fat depot in the surrounding atria with endocrine and inflammatory properties, EAT is also implicated in the pathophysiology of AF^[11]^. Thus, there are several studies demonstrating the relationship of EAT with POAF. For example, it was previously demonstrated that pericardial fat volume was measured by computed tomography (CT) in 83 patients who underwent CABG surgery and can strongly predict the POAF development regardless of other factors^[6]^. Kogo et al.^[12]^ investigated EAT thickness with CT in 77 patients who underwent cardiac surgery and found left atrial EAT/total EAT ratio was shown to be strongly associated with POAF development. Romanov et al.^[13]^ showed the relationship between EAT and POAF in an *in-vivo* study in which botulinum toxin was injected into the epicardial fat tissue in patients undergoing cardiac surgery and heart rhythms were followed by an implantable cardiac monitor for three years. AF attacks were significantly lower in patients who underwent botulinum injection.

All these studies show that EAT plays a central role in the development of POAF. Our results are consistent with the studies mentioned above. Although both groups were similar according to baseline clinical and demographic data, we found that the mean EAT thickness, non-invasively measured by TTE, was significantly higher in POAF patients. We have also shown that EAT thickness independently and strongly predicts POAF development. Moreover, we showed that EAT thickness above 7.05 mm can predict POAF development in ROC analysis. Unlike the aforementioned studies, we evaluated the relationship between EAT thickness measured by TTE, which is a non-invasive, easier and more reproducible method than CT. Thus, CT has some disadvantages including the use of intravenously administered iodinated contrast material and exposure to ionizing radiation. In addition, if CT is performed without ECG gating or triggering, as is often the case, fat thickness in the image can differ according to the cardiac cycle; thus, cardiac motion artifacts can limit the evaluation of the pericardium^[14]^. Therefore, routine use of CT cannot be recommended for this purpose. In this regard, the result of our study is important since EAT measurement during preoperative routine echocardiographic examination may easily provide an important clue about which patients are at high risk for developing POAF.

The relationship of EAT with AF development is not a surprising and has several mechanisms^[11]^. One of them is myocardial cell damage, an advanced remodeling secondary to impaired reninangiotensin-aldosterone system, oxidative stress and hypoxia^[15]^. Formations of reactive oxygen species (ROS) are involved in the development of AF. On the other hand, NADPH-induced production of ROS has been demonstrated in human EAT and the ability to generate ROS of EAT is significantly greater than subcutaneous adipose tissue^[16]^. EAT is also a metabolically active tissue, its secretions, as a paracrine effect, can directly affect the atrium due to the anatomical neighborhood^[17]^. Activin A, one of the substances secreted by EAT, shows profibrotic effects on the atrium and increases the risk of AF^[18]^. Moreover, EAT may probably enhances atrial myocardial inflammation via cytokines such as IL6, IL-1B, IL-8, TNF-alfa and facilitates AF development^[19]^. Therefore, an increased EAT may have an increased risk of new-onset AF in the postoperative period due to EAT associated inflammatory status. In addition, we found a positive correlation between EAT and LAVI. Recent studies confirm that pericardial fat is associated with LA dimensions^[20]^. Adipose tissue-related mediators can also cause increased inflammatory activation in the atrial wall and in the myocardial wall. Such cytokines can cause atrial remodeling, such as enlarging of atriums and can also cause AF indirectly. Thus, there is compelling evidence of the local effect of epicardial and pericardial fat in the pathogenesis of AF, possibly through fatty atrial infiltration, fibrosis, and inflammation^[21]^. On the other hand, investigators tried to explain the mechanism of how pericardial fat affects left atrial dilatation. However, the pathogenic mechanism of these alterations is not well known yet. There is strong evidence that left atrial enlargement, including adiposity, correlates with an increased AF risk. Thus, EAT may induce POAF directly via increased local inflammation and also indirectly by causing myocardial remodeling.

In the current study, we found that patients with POAF had longer CC and CPB times than Non-AF group patients. In addition to preoperative factors, some intraoperative factors have been suggested to predict the risk of postoperative AF after CABG. Although CPB procedure is still the gold standard in patients with multivessel coronary artery disease undergoing CABG, it increases the systemic inflammatory response. Thus, inflammation is one of the factors that increase the risk of POAF, as noted above^[3]^. It has been previously reported that inflammatory markers such as IL-1, IL-6, IL-8 and C-reactive protein were found to be elevated after CPB^[22]^. Thus, longer CPB and CC times means higher systemic inflammation, increasing the risk of POAF. Inflammatory theory on the development of AF was also supported by another finding that we showed the use of statin predicted POAF development. In fact, it was reported that statin treatment reduces the risk of POAF development in a previously published meta-analysis^[23]^. Presumably, statins reduce the risk of AF development with their anti-inflammatory effects. Interestingly, we also found that EAT thickness was positively correlated with both CC and CPB times and both can predict the POAF occurrence in POAF groups in the regression analysis. It may be also explained by adipose tissue-related inflammation. An increased EAT may cause prolonged cardioplegia. Indeed, it was previously demonstrated that the magnitude of the IL-6 response, observed from the interval just before anesthesia induction until sternal closure, is correlated with the duration of CPB^[24]^. Also, an excess of epicardial fat would make more difficult and time-consuming to find the coronary artery targets for bypass and to perform the anastomoses.

Finally, we found significantly higher NT-proBNP levels in patients developing POAF. Regression analysis showed that NTproBNP levels predict POAF development in the POAF group. As a well-known marker in the diagnosis and management of heart failure, NT-proBNP reflects left ventricle pressure and volume load. In OPERA trial, it was found that BNP and NT-proBNP levels were associated with increased risk of POAF^[25]^. The results of our study were similar to previous studies. An increase in left ventricular pressure may trigger POAF through structural and electrical changes in the left atrium.

### Limitation

The major limitation of this study is the limited number of patients studied. Our findings must be supported by studies involving a larger population. In addition, we could evaluate systemic inflammatory markers to support the study results.

## CONCLUSION

According to the results of the current study, a preoperative measurement of EAT thickness prior to cardiac surgery may be included in routine echocardiographic parameters to determine which patients are at high risk of developing POAF.

**Table:** 

Authors' roles & responsibilities	
EEG	Substantial contributions to the conception or design of the work; or the acquisition, analysis, or interpretation of data for the work; drafting the work or revising it critically for important intellectual content; final approval of the version to be published
MT	Substantial contributions to the conception or design of the work; or the acquisition, analysis, or interpretation of data for the work; drafting the work or revising it critically for important intellectual content; final approval of the version to be published
FS	Substantial contributions to the conception or design of the work; or the acquisition, analysis, or interpretation of data for the work; drafting the work or revising it critically for important intellectual content; final approval of the version to be published
HA	Substantial contributions to the conception or design of the work; or the acquisition, analysis, or interpretation of data for the work; drafting the work or revising it critically for important intellectual content; final approval of the version to be published

## References

[1] Rostagno C, La Meir M, Gelsomino S, Ghilli L, Rossi A, Carone E (2010). Atrial fibrillation after cardiac surgery: incidence, risk factors, and economic burden. J Cardiothorac Vasc Anesth.

[2] Horwich P, Buth KJ, Légaré JF (2013). New onset postoperative atrial fibrillation is associated with a long-term risk for stroke and death following cardiac surgery. J Card Surg.

[3] Jakubová M, Mitro P, Stančák B, Sabol F, Kolesár A, Cisarik P (2012). The occurrence of postoperative atrial fibrillation according to different surgical settings in cardiac surgery patients. Interact Cardiovasc Thorac Surg.

[4] Sacks HS, Fain JN (2007). Human epicardial adipose tissue: a review. Am Heart J.

[5] Bohatch Júnior MS, Matkovski PD, Di Giovanni FJ, Fenili R, Varella EL, Dietrich A (2015). Incidence of postoperative atrial fibrillation in patients undergoing onpump and off pump coronary artery bypass grafting. Rev Bras Cir Cardiovasc.

[6] Drossos G, Koutsogiannidis CP, Ananiadou O, Kapsas G, Ampatzidou F, Madesis A (2014). Pericardial fat is strongly associated with atrial fibrillation after coronary artery bypass graft surgery†. Eur J Cardiothorac Surg.

[7] Burstein B, Nattel S (2008). Atrial fibrosis: mechanisms and clinical relevance in atrial fibrillation. J Am Coll Cardiol.

[8] Venteclef N, Guglielmi V, Balse E, Gaborit B, Cotillard A, Atassi F (2015). Human epicardial adipose tissue induces fibrosis of the atrial myocardium through the secretion of adipo-fibrokines. Eur Heart J.

[9] Iacobellis G, Assael F, Ribaudo MC, Zappaterreno A, Alessi G, Di Mario U (2003). Epicardial fat from echocardiography: a new method for visceral adipose tissue prediction. Obes Res.

[10] Kaireviciute D, Aidietis A, Lip GY (2009). Atrial fibrillation following cardiac surgery: clinical features and preventative strategies. Eur Heart J.

[11] Aksoy F, Guler S, Kahraman F, Oskay T, Varol E (2019). The relation between echocardiographic epicardial fat thickness and CHA2DS2-VASc score in patients with sinus rhythm. Braz J Cardiovasc Surg.

[12] Kogo H, Sezai A, Osaka S, Shiono M, Tanaka M (2019). Does epicardial adipose tissue influence postoperative atrial fibrillation?. Ann Thorac Cardiovasc Surg.

[13] Romanov A, Pokushalov E, Ponomarev D, Bayramova S, Shabanov V, Losik D (2019). Long-term suppression of atrial fibrillation by botulinum toxin injection into epicardial fat pads in patients undergoing cardiac surgery: three-year follow-up of a randomized study. Heart Rhythm.

[14] Wang ZJ, Reddy GP, Gotway MB, Yeh BM, Hetts SW, Higgins CB (2003). CT and MR imaging of pericardial disease. Radiographics.

[15] Kim YM, Guzik TJ, Zhang YH, Zhang MH, Kattach H, Ratnatunga C (2005). A myocardial Nox2 containing NAD(P)H oxidase contributes to oxidative stress in human atrial fibrillation. Circ Res.

[16] Salgado-Somoza A, Teijeira-Fernández E, Fernández AL, González-Juanatey JR, Eiras S (2010). Proteomic analysis of epicardial and subcutaneous adipose tissue reveals differences in proteins involved in oxidative stress. Am J Physiol Heart Circ Physiol.

[17] Iacobellis G, Corradi D, Sharma AM (2005). Epicardial adipose tissue: anatomic, biomolecular and clinical relationships with the heart. Nat Clin Pract Cardiovasc Med.

[18] Wang Q, Min J, Jia L, Xi W, Gao Y, Diao Z (2019). Human epicardial adipose tissue activin A expression predicts occurrence of postoperative atrial fibrillation in patients receiving cardiac surgery. Heart Lung Circ.

[19] Echahidi N, Pibarot P, O'Hara G, Mathieu P (2008). Mechanisms, prevention, and treatment of atrial fibrillation after cardiac surgery. J Am Coll Cardiol.

[20] Fox CS, Gona P, Hoffmann U, Porter SA, Salton CJ, Massaro JM (2009). Pericardial fat, intrathoracic fat, and measures of left ventricular structure and function: the Framingham heart study. Circulation.

[21] Batal O, Schoenhagen P, Shao M, Ayyad AE, Van Wagoner DR, Halliburton SS (2010). Left atrial epicardial adiposity and atrial fibrillation. Circ Arrhythm Electrophysiol.

[22] Elgendy IY, Mahmoud A, Huo T, Beaver TM, Bavry AA (2015). Meta-analysis of 12 trials evaluating the effects of statins on decreasing atrial fibrillation after coronary artery bypass grafting. Am J Cardiol.

[23] Nakai T, Lee RJ, Schiller NB, Bellows WH, Dzankic S, Reeves J 3rd (2002). The relative importance of left atrial function versus dimension in predicting atrial fibrillation after coronary artery bypass graft surgery. Am Heart J.

[24] Whitten CW, Hill GE, Ivy R, Greilich PE, Lipton JM (1998). Does the duration of cardiopulmonary bypass or aortic cross-clamp, in the absence of blood and/or blood product administration, influence the IL-6 response to cardiac surgery?. Anesth Analg.

[25] Masson S, Wu JH, Simon C, Barlera S, Marchioli R, Mariani J (2015). Circulating cardiac biomarkers and postoperative atrial fibrillation in the OPERA trial. Eur J Clin Invest.

